# Association of dietary intake patterns with treatment outcomes in late-stage pancreatic tumors

**DOI:** 10.3389/fnut.2026.1757545

**Published:** 2026-04-13

**Authors:** Wei Cui, Yong Tao, Sai Zhang, Wei Zhou, ZhiHua Liu

**Affiliations:** Department of General Surgery, Wannan Medical College Affiliated Xuancheng Hospital, Xuancheng City People's Hospital, Xuancheng, China

**Keywords:** dietary intake patterns, late-stage pancreatic tumors, nutritional status, pancreatic cancer, survival outcomes, treatment outcomes

## Abstract

**Background:**

Dietary intake patterns are increasingly recognized as potential factors associated with treatment response and survival in patients with advanced pancreatic cancer; however, evidence from real-world clinical settings remains limited.

**Methods:**

This retrospective observational study analyzed electronic medical record data from 210 patients with Stage III–IV pancreatic adenocarcinoma. Dietary patterns were categorized as High-Fat Western, Mixed/Transitional, or Plant-Rich based on standardized nutritional assessments. Clinical characteristics, treatment regimens, nutrient intake, chemotherapy response, progression-free survival (PFS), overall survival (OS), and adverse events were observed. Multivariate logistic regression and Cox proportional hazards models were used to examine the independent association between dietary factors and treatment outcomes.

**Results:**

Significant differences in nutrient profiles were observed across dietary groups (*p* < 0.01). The High-Fat Western group had the highest intake of saturated fat, cholesterol, and processed meats, along with the lowest intake of fiber and micronutrients. The dietary pattern was associated with lower rates of complete/partial response (15.8%), shorter OS (6.4 ± 2.1 months), and shorter PFS (3.2 ± 1.5 months). In contrast, the plant-rich pattern was associated with the most favorable nutrient intake and longer survival outcome (OS 10.5 ± 3.2 months; PFS 5.3 ± 2.0 months). Multivariable analyses demonstrated that adherence to High-Fat Western diets was associated with increased odds of progressive disease (OR = 2.96; 95% CI: 1.59–5.48) and higher hazards of death or progression (HR = 2.42; 95% CI: 1.57–3.74). Low fiber intake, low omega-3, high saturated fat, and lower vitamin C were independently associated with poor outcomes. Additionally, clinical complications, such as severe fatigue, cachexia progression, dose reductions, and treatment discontinuation, were significantly associated with reduced survival.

**Conclusion:**

In patients with advanced pancreatic cancer, diet quality was significantly associated with treatment response and survival outcomes. Plant-rich dietary patterns were associated with more favorable clinical outcomes, whereas high-fat, processed-food dietary patterns were associated with poorer response rates and shorter survival. These findings highlight a clinically meaningful association between nutritional status and oncologic outcomes and support the consideration of routine nutritional assessment.

## 1 Introduction

Pancreatic cancer is the seventh biggest cause of cancer fatalities and has a five-year survival rate of fewer than 10%. Its prevalence is rising worldwide, especially in industrializing countries. Nearly one-third of global pancreatic cancer cases are in China (1). Japanese, South Korean, and Indian lifestyle changes, urbanization, and ageing populations have also led to rising frequency (2). These regions have many late-stage diagnoses due to the disease’s quiet progression and absence of early screening techniques. Most patients have advanced or metastatic tumors, limiting treatment options and lowering therapeutic response rates (3, 4). Asian pancreatic cancer rates are rising due to multiple risk factors. The region has high rates of smoking, obesity, chronic pancreatitis, familial history, and type-II diabetes (5). Rapid diet changes have increased ultra-processed, high-calorie, and high-fat meals and decreased fiber-rich fruits, vegetables, and whole grains. These changes are occurring alongside Westernized diets heavy in saturated fats, cholesterol, refined carbohydrates, and red or processed meats (6, 7). Diet-induced chronic inflammation, oxidative stress, insulin resistance, and gut microbial dysbiosis can cause cancers (8). Diet affects pancreatic tumor biological pathways (9). Consuming more saturated fats increases cytokine production, particularly TNF-*α* and IL-6, and activates the NF-κB pathway, promoting new blood vessel growth. These traits promote tumor growth and treatment resistance (10). Low fiber, antioxidant-poor, and low omega-3 dietary patterns are associated with impaired gut integrity, increased inflammation and oxidative stress, and altered tumor-related metabolic and signaling pathways (11–13). Therefore, a retrospective analysis using electronic medical record data is needed to evaluate the association between dietary intake patterns and clinical outcomes within a real-world oncology setting. Such an approach allows integration of nutritional assessments with treatment regimens, complications, and survival endpoints to better clarify whether diet quality is linked to therapeutic response and prognosis. According to this study, nutrition may help or hinder treatment. By addressing these gaps, this study aims to provide clinically relevant evidence regarding the association between dietary patterns and outcomes in late-stage pancreatic cancer.

## 2 Materials and methods

### 2.1 Study design and data source

The researchers conducted a retrospective observational study using a tertiary cancer center’s EMR system to obtain patient data. All clinical, biochemical, therapeutic, and nutritional information was extracted from the patient’s digital records. The dataset included patient demographics, tumor data, comorbidities, laboratory indicators, treatment protocols, adverse events, and follow-up results from the patient’s file. Patients’ dietary intake was assessed using nutrition evaluation questionnaires completed at diagnosis and updated during therapy, recorded in a structured case proforma. The proforma was reviewed by experts for content validity and tested for reliability to ensure consistency and accuracy of the collected data.

### 2.2 Participant selection

All 250 patients tested had advanced pancreatic adenocarcinoma (Stage III–IV). Predetermined inclusion and exclusion criteria were used to assess study eligibility. The inclusion criteria were a histologically confirmed pancreatic adenocarcinoma diagnosis, stage III or IV disease at diagnosis, comprehensive dietary assessment records, and at least one cycle of systemic therapy. Lack of evidence about nutritional intake or treatment, concurrent malignancies, significant hepatic or renal failure unrelated to the condition, and enrolment in experimental or unblinded nutritional intervention studies were disqualifying factors. All 210 patients who applied met the qualifying criteria in the final analysis.

### 2.3 Dietary pattern assessment and treatment data

Dietary intake data were obtained from patients’ electronic medical records, where routine nutritional assessments were conducted by clinical dietitians at the time of diagnosis using a semi-quantitative dietary questionnaire and a 24-h dietary recall template. The assessments documented the frequency and portion sizes of major food groups and were reviewed by physicians and dietitians before being stored in the EMR system. For this retrospective analysis, the baseline dietary information recorded at diagnosis prior to chemotherapy was extracted and used to classify dietary patterns. Three primary eating patterns were found using preset criteria: The high-fat western diet includes saturated fats, processed meals, red/processed meats, sugary drinks, and few fruits, vegetables, and whole grains. Mixed Diet: Moderate plant- and animal-based food consumption without any dominant dietary components. A plant-rich diet includes vegetables, fruits, whole grains, legumes, nuts, omega-3 s, and limited amounts of processed and high-fat animal products. EMR-based nutritional evaluations calculated daily nutrient intake, including fiber, omega-3, saturated fat, vitamin C, magnesium, and potassium, using meal frequency and 24-h recall templates. Oncology treatment records captured detailed information, including chemotherapy prescriptions, regimen types, dose modifications/intensity, treatment delays, adverse events, and response assessments. Treatment outcomes included tumor burden, progression-free survival (PFS), overall survival (OS), ECOG performance status, baseline CA19-9 levels, and chemotherapeutic response, categorized as complete response, partial response, stable disease, or disease progression. Additional clinical factors considered in the analysis included.

### 2.4 Statistical analysis

Data were analyzed using SPSS (version 26). Categorical variables were expressed as frequencies and percentages and compared using chi-square tests. Continuous variables were summarized as mean ± SD or median (IQR) and analyzed using ANOVA. Multivariate logistic regression was used to identify independent predictors of poor treatment response, adjusting for age, sex, BMI, tumor stage, comorbidities, and dietary factors. Hazard ratios for OS and PFS were assessed using Cox proportional hazards models. A significance level of *p* < 0.05 was considered statistically significant.

### 2.5 Ethical consideration

This research was approved by the Ethics Committee of Wannan Medical College Affiliated Xuancheng Hospital. Ethics Approval No.2023-1w012-01. All techniques followed the Declaration of Helsinki (2013 revision) ethics. We waived informed consent for this retrospective study. Anonymous records and restricted access to authorized staff protected patient data.

## 3 Results

### 3.1 Demographic and clinical characteristics of the study population

The present study included 210 people with advanced pancreatic tumors. Patients had three dietary consumption patterns: High-Fat Western (*n* = 76), Mixed/Transitional (*n* = 69), and Plant-Rich (*n* = 65). Table 1 presents the demographic and clinical variables across these groups. The mean age was 63.4 ± 9.8 years, with 46.7% aged ≥65 years. The distribution of age did not differ significantly across dietary groups (*p* = 0.172). Males comprised 61% of the cohort, with a similar sex distribution between groups (*p* = 0.621). BMI differed significantly across dietary groups (*p* < 0.001), highest in the High-Fat Western group (26.1 ± 4.1 kg/m^2^) and lowest in the Plant-Rich group (23.4 ± 3.2 kg/m^2^). Current smoking was 30.3, 21.7, and 20.0% in High-Fat Western, Mixed, and Plant-Rich groups, respectively (*p* = 0.087). Stage IV disease was present in 62.9% of patients, with no significant difference between groups (*p* = 0.204). Diabetes prevalence was 55.3% in the High-Fat Western group, 44.9% in Mixed, and 36.9% in Plant-Rich (*p* = 0.053), while hypertension prevalence was 50.0, 42.0, and 32.3%, respectively (*p* = 0.061). Chronic pancreatitis and family history of cancer were reported across groups with no significant difference (*p* = 0.182 and *p* = 0.194).

**Table 1:** Demographic and clinical characteristics of participants.

Variable	Total *n* (%)	High-fat western (*n* = 76)	Mixed/transitional (*n* = 69)	Plant-rich (*n* = 65)	*p*-value
Age (years), mean ± SD	63.4 ± 9.8	64.8 ± 8.7	63.1 ± 10.2	61.9 ± 9.6	0.184
Age ≥65 years	98 (46.7)	41 (53.9)	32 (46.4)	25 (38.5)	0.172
Sex					0.621
Male	128 (61.0)	49 (64.5)	41 (59.4)	38 (58.5)	
Female	82 (39.0)	27 (35.5)	28 (40.6)	27 (41.5)	
BMI (kg/m^2^), mean ± SD	24.7 ± 3.9	26.1 ± 4.1	24.4 ± 3.7	23.4 ± 3.2	<0.001
Smoking status					0.087
Never	92 (43.8)	26 (34.2)	31 (44.9)	35 (53.8)	
Former	67 (31.9)	27 (35.5)	23 (33.3)	17 (26.2)	
Current	51 (24.3)	23 (30.3)	15 (21.7)	13 (20.0)	
Stage of tumor					0.204
Stage III	78 (37.1)	23 (30.3)	26 (37.7)	29 (44.6)	
Stage IV	132 (62.9)	53 (69.7)	43 (62.3)	36 (55.4)	
Comorbidities					
Diabetes	97 (46.2)	42 (55.3)	31 (44.9)	24 (36.9)	0.053
Hypertension	88 (41.9)	38 (50.0)	29 (42.0)	21 (32.3)	0.061
Chronic pancreatitis	32 (15.2)	15 (19.7)	11 (15.9)	6 (9.2)	0.182
Family history of cancer	44 (21.0)	19 (25.0)	16 (23.2)	9 (13.8)	0.194

Values are presented as mean ± standard deviation or *n* (%). *p*-values were derived from ANOVA for continuous variables and chi-square tests for categorical variables.

### 3.2 Dietary intake patterns and their nutritional differences among groups

Table 2 presents the comparisons of macronutrient and micronutrient intake across three dietary groups: High-Fat Western (*n* = 76), Mixed/Transitional (*n* = 69), and Plant-Rich (*n* = 65). Significant differences were observed in total energy and nutrient consumption across the groups (*p* < 0.01 for most variables). Participants in the High-Fat Western diet group had the highest total energy intake (2,450 ± 320 kcal/day), fat (96 ± 18 g/day), saturated fat (34 ± 7 g/day), cholesterol (310 ± 68 mg/day), and red and processed meats (145 ± 32 g/day). The Mixed/Transitional group had intermediate values for these macronutrients, while the Plant-Rich group had the lowest values. Fiber and plant-based foods varied across groups, with the Plant-Rich group consuming the highest fiber (26 ± 6 g/day), fruit (3.2 ± 1.1 servings/day), and vegetables (4.0 ± 1.2 servings/day). The Mixed/Transitional group showed intermediate intake (fiber 18 ± 5 g/day, fruits 1.8 ± 0.7 servings/day, vegetables 2.3 ± 0.9 servings/day), and the High-Fat Western group had the lowest intake. Micronutrient intake also differed between groups. The Plant-Rich group had the highest intake of vitamin A (910 ± 210 μg/day), vitamin C (123 ± 34 mg/day), vitamin E (13.6 ± 3.1 mg/day), calcium (910 ± 184 mg/day), magnesium (300 ± 63 mg/day), potassium (3,450 ± 520 mg/day), and omega-3 fatty acids (1.3 ± 0.4 g/day). The Mixed/Transitional group had intermediate values, and the High-Fat Western group had the lowest values for these micronutrients. Zinc intake was slightly higher in the High-Fat Western group (9.2 ± 1.4 mg/day) compared with the other two groups (Table 2).

**Table 2:** Dietary patterns: macronutrient and micronutrient consumption.

Nutrient/intake	High-fat western (*n* = 76)	Mixed/transitional (*n* = 69)	Plant-rich (*n* = 65)	*p*-value
Total energy (kcal/day)	2,450 ± 320	2,100 ± 290	1850 ± 270	<0.001
Carbohydrates (g/day)	280 ± 45	260 ± 41	240 ± 39	0.002
Protein (g/day)	88 ± 15	82 ± 14	76 ± 12	0.009
Total fat (g/day)	96 ± 18	78 ± 17	60 ± 13	<0.001
Saturated Fat (g/day)	34 ± 7	26 ± 6	18 ± 5	<0.001
Fiber (g/day)	14 ± 4	18 ± 5	26 ± 6	<0.001
Cholesterol (mg/day)	310 ± 68	240 ± 56	160 ± 45	<0.001
Fruit intake (serv/day)	0.9 ± 0.5	1.8 ± 0.7	3.2 ± 1.1	<0.001
Vegetable intake (serv/day)	1.1 ± 0.6	2.3 ± 0.9	4.0 ± 1.2	<0.001
Red/processed meat (g/day)	145 ± 32	98 ± 28	52 ± 21	<0.001
Micronutrients				
Vitamin A (μg/day)	620 ± 140	730 ± 180	910 ± 210	<0.001
Vitamin C (mg/day)	58 ± 22	84 ± 27	123 ± 34	<0.001
Vitamin E (mg/day)	7.8 ± 1.9	10.1 ± 2.5	13.6 ± 3.1	<0.001
Calcium (mg/day)	640 ± 140	780 ± 162	910 ± 184	<0.001
Magnesium (mg/day)	180 ± 44	230 ± 51	300 ± 63	<0.001
Potassium (mg/day)	2,200 ± 360	2,800 ± 430	3,450 ± 520	<0.001
Zinc (mg/day)	9.2 ± 1.4	8.7 ± 1.5	8.1 ± 1.2	0.003
Omega-3 (g/day)	0.4 ± 0.2	0.8 ± 0.3	1.3 ± 0.4	<0.001

Values are presented as mean ± standard deviation. *p*-values were calculated using one-way ANOVA. No adjustment for multiple comparisons was applied, therefore, individual *p*-values should be interpreted cautiously.

### 3.3 Baseline clinical characteristics, treatment outcomes, and their association with dietary patterns

According to Table 3, baseline clinical characteristics and treatment outcomes differed across the three dietary patterns. The High-Fat Western diet group had the lowest proportion of complete or partial chemotherapeutic responses (15.8%) and the highest proportion of progressive disease (56.6%), compared with the Mixed/Transitional group (26.1 and 42.0%, respectively) and the Plant-Rich group (33.8 and 29.2%, respectively; *p* = 0.014), Overall survival (OS) was 6.4 ± 2.1 months in the High-Fat Western group, 8.1 ± 2.3 months in the Mixed group, and 10.5 ± 3.2 months in the Plant-Rich group (*p* < 0.001). Progression-free survival (PFS) was 3.2 ± 1.5 months, 4.1 ± 1.9 months, and 5.3 ± 2.0 months, respectively (p < 0.001). One-year survival rates were 14.5, 23.2, and 36.9% for the High-Fat Western, Mixed, and Plant-Rich groups, respectively (*p* = 0.006). ECOG performance status 0–1 was observed in 65.8, 69.6, and 80.0% of patients, and ECOG ≥2 in 34.2, 30.4, and 20.0%, respectively (*p* = 0.120). Mean baseline CA19-9 levels were 845 ± 210 U/mL, 790 ± 195 U/mL, and 720 ± 180 U/mL, respectively (*p* = 0.045). Tumor burden (≥2 metastatic sites) occurred in 36.8, 31.9, and 23.1% of patients, respectively (*p* = 0.110). Chemotherapy regimen distribution was as follows: FOLFIRINOX, 47.4, 49.3, and 53.8%; Gemcitabine/Nab-Paclitaxel, 52.6, 50.7, and 46.2%, respectively. Treatment-related adverse events showed higher incidences in the High-Fat Western group, including severe fatigue (63.2, 55.1, 44.6%; *p* = 0.041), cachexia progression (53.9, 37.7, 23.1%; *p* = 0.002), chemotherapy dose reduction (42.1, 31.9, 15.4%; *p* = 0.001), and treatment discontinuation (30.3, 20.3, 13.8%; *p* = 0.047). Episodes of infection and hospitalizations did not differ significantly across the groups (*p* = 0.220 and 0.178, respectively).

**Table 3:** Baseline clinical characteristics, treatment outcomes and their association with dietary patterns.

Outcome	High-fat western (*n* = 76)	Mixed (*n* = 69)	Plant-rich (*n* = 65)	*p*-value
Baseline clinical characteristics				
ECOG performance status 0–1	50 (65.8%)	48 (69.6%)	52 (80.0%)	0.120
ECOG ≥2	26 (34.2%)	21 (30.4%)	13 (20.0%)	
Baseline CA19-9 (U/mL), mean ± SD	845 ± 210	790 ± 195	720 ± 180	0.045
Tumor Burden (n ≥ 2 metastatic sites)	28 (36.8%)	22 (31.9%)	15 (23.1%)	0.110
Chemotherapy regimen: FOLFIRINOX	36 (47.4%)	34 (49.3%)	35 (53.8%)	0.810
Gemcitabine/Nab-Paclitaxel	40 (52.6%)	35 (50.7%)	30 (46.2%)	
Treatment outcomes				
Chemotherapy response				0.014
Complete/partial response	12 (15.8%)	18 (26.1%)	22 (33.8%)	
Stable disease	21 (27.6%)	22 (31.9%)	24 (36.9%)	
Progressive disease	43 (56.6%)	29 (42.0%)	19 (29.2%)	
Overall survival (months)	6.4 ± 2.1	8.1 ± 2.3	10.5 ± 3.2	<0.001
PFS (months)	3.2 ± 1.5	4.1 ± 1.9	5.3 ± 2.0	<0.001
1-year survival	11 (14.5%)	16 (23.2%)	24 (36.9%)	0.006
Severe fatigue	48 (63.2%)	38 (55.1%)	29 (44.6%)	0.041
Infection episodes	29 (38.2%)	22 (31.9%)	17 (26.1%)	0.220
Hospitalizations	31 (40.8%)	23 (33.3%)	18 (27.7%)	0.178
Cachexia progression	41 (53.9%)	26 (37.7%)	15 (23.1%)	0.002
Dose reduction	32 (42.1%)	22 (31.9%)	10 (15.4%)	0.001
Treatment discontinuation	23 (30.3%)	14 (20.3%)	9 (13.8%)	0.047

Values are presented as *n* (%) or mean ± SD. *p*-values were calculated using chi-square tests for categorical outcomes and one-way ANOVA for continuous outcomes.

### 3.4 Predictors of treatment response and survival in late-stage pancreatic cancer

The results of the combined logistic and Cox regression analyses for 210 patients with pancreatic tumors are presented in Table 4. Compared to the Plant-Rich reference group, patients who followed a High-Fat Western diet had a considerably greater chance of experiencing progressive illness (odds ratio = 2.96; 95 percent confidence interval: 1.59–5.48; *p* = 0.001) as well as a significantly increased hazard of advancement or death (hazard ratio = 2.42; 95 percent confidence interval: 1.57–3.74; *p* < 0.001), (Table 4). On the other hand, when compared to the other diets, the Mixed diet exhibited trends that were not statistically significant in any model. These trends were associated with poorer results. There was an independent association observed between nutritional deficiencies and a greater likelihood of progression, as well as a shorter survival time. These deficiencies included a low intake of fiber (less than 15 grammes per day), a low intake of omega-3 fatty acids (less than 0.6 grammes per day), a high intake of saturated fats (more than 30 grammes per day), and a low intake of vitamin C (less than 60 milligrams per day). The odds ratios for these deficiencies ranged from 1.77 to 2.39, and the *p*-values were less than 0.05. The hazard ratios for these deficiencies ranged from 1.52 to 1.91, and the p-values were less than or equal to 0.038. Another significant predictor of both poor response and shorter survival was the presence of clinical and treatment-related variables, including the advancement of cachexia, the lowering of chemotherapy doses, and the termination of treatment, as well as the presence of severe tiredness. The advanced stage of the tumor (Stage IV) continued to be one of the most significant indicators that were present in both analyses (OR = 2.34; HR = 2.05; *p* ≤ 0.006).

**Table 4:** Combined logistic and Cox regression analysis: predictors of poor treatment response and survival in late-stage pancreatic cancer.

Variable	Logistic regression OR (95% CI)	*p*-value	Cox regression HR (95% CI)	*p*-value
Age ≥65 years	1.41 (0.87–2.28)	0.164	1.21 (0.89–1.64)	0.210
Male sex	1.22 (0.73–2.04)	0.445	1.12 (0.82–1.53)	0.470
BMI ≥ 25 kg/m^2^	1.51 (0.90–2.53)	0.116	1.28 (0.94–1.74)	0.110
Stage IV tumor	2.34 (1.27–4.32)	0.006	2.05 (1.35–3.10)	0.001
Diabetes	1.37 (0.85–2.22)	0.188	1.18 (0.85–1.64)	0.310
Hypertension	1.18 (0.72–1.94)	0.515	1.10 (0.78–1.55)	0.580
High-fat western diet	2.96 (1.59–5.48)	0.001	2.42 (1.57–3.74)	<0.001
Mixed diet	1.52 (0.83–2.77)	0.171	1.45 (0.93–2.27)	0.100
Plant-rich diet (ref)	—	—	—	—
Low fiber (<15 g/day)	2.04 (1.18–3.53)	0.011	1.78 (1.14–2.79)	0.011
Low Omega-3 (<0.6 g/day)	1.88 (1.07–3.30)	0.029	1.63 (1.03–2.57)	0.038
High saturated fat (>30 g/day)	2.39 (1.31–4.36)	0.004	1.91 (1.20–3.03)	0.006
Low Vitamin C (<60 mg/day)	1.77 (1.02–3.07)	0.042	1.52 (0.96–2.40)	0.075
Severe fatigue	1.70 (1.04–2.77)	0.034	1.61 (1.05–2.47)	0.028
Cachexia progression	2.50 (1.39–4.49)	0.002	2.05 (1.33–3.15)	0.001
Chemotherapy dose reduction	1.94 (1.14–3.32)	0.015	1.69 (1.11–2.57)	0.015
Treatment Discontinuation	2.19 (1.22–3.92)	0.008	1.85 (1.16–2.95)	0.010

Logistic regression models the odds of progressive disease versus non-progressive disease; values are presented as adjusted odds ratios (OR) with 95% confidence intervals (CI). Cox proportional hazards regression models time-to-event outcomes (progression-free and overall survival); values are presented as hazard ratios (HR) with 95% CI. Reference categories: Plant-rich diet, absence of comorbidities or treatment-related complications, and age <65 years. Statistically significant associations are indicated by *p* < 0.05.

## 4 Discussion

Advanced pancreatic cancer patients’ food intake habits were associated with differences in their treatment responses (11). Clinical results have often been reported to differ between individuals consuming nutrient-dense or anti-inflammatory dietary patterns and those consuming more pro-inflammatory dietary patterns. There is growing evidence that pancreatic cancer patients’ metabolic and clinical profiles are associated with their diets. Western-style high-fat diets have been associated with higher levels of inflammation and metabolic stress (14), whereas plant-based diets are characterized by more antioxidants, less saturated fat, and more fiber consumption, which have been associated with improved cardiometabolic profiles and lower levels of inflammatory markers. The contrast of these patterns has been linked to differences in long-term health (2, 15). The present study’s findings are consistent with earlier research, supporting the association between baseline health status in this population and dietary quality. A prior study found that Western eaters had a higher body mass index and more metabolic comorbidities, similar to our High-Fat Western group (14, 16). Another study presented a link between plant-based diets and lower in smoking and hypertension rates (17), which was observed in our Plant-Rich group, which had the fewest smokers and hypertensives. According to a previous study, the High-Fat Western group was associated with higher rates of diabetes (18). Plant-rich diets have been linked to lower BMI and fewer metabolic comorbidities (19), which is consistent with our Plant-Rich participants’ lower BMI and diabetes and hypertension burdens. A growing number of studies demonstrate an association between dietary exposures and pancreatic tumor biology and pancreatic tumor biology and inflammatory signaling pathways. Another study found that higher intake of saturated fats and cholesterol was linked to increased TNF-*α* and IL-6 activity in the pancreatic cancer microenvironment (20, 21), which is consistent with the Western diet intake observed in our sample. Low fiber intake has been associated with gut microbiome dysbiosis and reduced short-chain fatty acid (SCFA) production (22), which is consistent with Western diets’ low fiber intake (14 g/day). High red and processed meat consumption has been linked to increased nitrosamine exposure and oxidative stress (23), reflecting our Western diet cohort.

The Plant-Rich group’s diets were associated with higher intake of fruits, vegetables, and vitamin C, which in previous studies have been associated with differences in oxidative stress markers and cellular signaling pathways (24). Higher magnesium and potassium consumption was observed in the plant group, consistent with the findings linking the nutrients to improve glucose regulation and reduce metabolic stress (25). Increased omega-3 consumption in the Plant-Rich group was reported in previous studies to be associated with differences in chemotherapy response and inflammatory signaling pathways (26).

Western diets, high in saturated fat, cholesterol, processed meats, and low fiber, have been linked to increased inflammatory signaling, oxidative stress, and microbiome disruption. Plant-rich diets have been associated with metabolic, anti-oxidative, and anti-inflammatory effects. High-fiber intake was associated with improved survival and reduced systemic inflammation (27, 28), consistent with higher overall and progression-free survival in the Plant-Rich group. Higher red or processed meat intake was also associated with oxidative stress and altered metabolism, which have been linked to differences in chemotherapy outcomes (29). Antioxidant-rich diets were associated with fewer adverse effects and improved immunological competence after chemotherapy (6, 7), similar to the Plant-Rich cohort, which had less fatigue and fewer dose modifications. Vitamin C deficiency has been linked to reduced antioxidant capacity and differences in therapy response reported in earlier research (30, 31), consistent with our study.

Overall, this study supports the association reported in previous research between dietary patterns, nutritional status, and treatment factors and chemotherapy response and survival outcomes in advanced-stage pancreatic cancer patients. Plant-rich, nutrient-dense diets were associated with more favorable clinical profiles, whereas high-fat, low-fiber, nutrient-poor diets were associated with less favorable outcomes.

## 5 Limitations

This study has limitations that should be considered when interpreting the findings. First, the retrospective observational design limits the ability to establish causal relationships between dietary patterns and treatment outcomes. Data were obtained from electronic medical records, and although multivariable models adjusted for major clinical factors, residual confounding from unmeasured variables such as socioeconomic status, physical activity, or detailed nutritional history cannot be completely excluded. Second, dietary intake information was derived from semi-quantitative dietary questionnaires and 24-h recall records documented in clinical practice. Such self-reported dietary assessments are inherently subject to recall bias, reporting inaccuracies, and potential misclassification of dietary patterns, which may affect the precision of nutrient intake estimates. Finally, baseline differences among dietary groups, including variations in BMI and prevalence of metabolic comorbidities, may have influenced clinical outcomes despite statistical adjustment. Therefore, while the findings demonstrate significant associations between diet quality and treatment outcomes, prospective cohort studies and controlled interventional trials are required to validate these observations and clarify potential causal mechanisms.

## 6 Conclusion

This study demonstrates that dietary patterns are associated with clinical outcomes in patients with late-stage pancreatic cancer. Patients consuming diets high in saturated fats and processed foods, with lower fiber and micronutrient intake, had lower treatment response rates, higher disease progression, and shorter overall and progression-free survival. In contrast, those following plant-based, nutrient-dense diets showed higher treatment response rates and longer survival. These findings reflect observed associations and do not establish causality. Further prospective studies are warranted to evaluate whether modifying dietary patterns can influence clinical outcomes in this population.

## Data Availability

The original contributions presented in the study are included in the article/supplementary material, further inquiries can be directed to the corresponding author.
